# Synergistic Antiproliferation of Cisplatin and Nitrated [6,6,6]Tricycle Derivative (SK2) for a Combined Treatment of Oral Cancer Cells

**DOI:** 10.3390/antiox11050926

**Published:** 2022-05-08

**Authors:** Sheng-Chieh Wang, Ching-Yu Yen, Jun-Ping Shiau, Meng-Yang Chang, Ming-Feng Hou, Jiiang-Huei Jeng, Jen-Yang Tang, Hsueh-Wei Chang

**Affiliations:** 1 Ph.D. Program in Life Sciences, Department of Biomedical Science and Environmental Biology, College of Life Sciences, Kaohsiung Medical University, Kaohsiung 80708, Taiwan; u107851101@gap.kmu.edu.tw (S.-C.W.); mifeho@kmu.edu.tw (M.-F.H.); 2 Department of Oral and Maxillofacial Surgery, Chi-Mei Medical Center, Tainan 71004, Taiwan; ycy@tmu.edu.tw; 3 School of Dentistry, Taipei Medical University, Taipei 11031, Taiwan; 4 Division of Breast Oncology and Surgery, Department of Surgery, Kaohsiung Medical University Hospital, Kaohsiung Medical University, Kaohsiung 80708, Taiwan; drshiaoclinic@gmail.com; 5 Department of Surgery, Kaohsiung Municipal Siaogang Hospital, Kaohsiung 81267, Taiwan; 6 Department of Medicinal and Applied Chemistry, Kaohsiung Medical University, Kaohsiung 80708, Taiwan; mychang@kmu.edu.tw; 7 School of Dentistry, College of Dental Medicine, Kaohsiung Medical University, Kaohsiung 80708, Taiwan; jhjeng@kmu.edu.tw; 8 Department of Dentistry, Kaohsiung Medical University Hospital, Kaohsiung 80708, Taiwan; 9 Department of Dentistry, National Taiwan University Hospital, Taipei 100225, Taiwan; 10 School of Post-Baccalaureate Medicine, Kaohsiung Medical University, Kaohsiung 80708, Taiwan; 11 Department of Radiation Oncology, Kaohsiung Medical University Hospital, Kaoshiung Medical University, Kaohsiung 80708, Taiwan; 12 Institute of Medical Science and Technology, National Sun Yat-sen University, Kaohsiung 80424, Taiwan; 13 Center for Cancer Research, Kaohsiung Medical University, Kaohsiung 80708, Taiwan

**Keywords:** nitrated [6,6,6]tricycles, combined treatment, antiproliferation, oral cancer

## Abstract

SK2, a nitrated [6,6,6]tricycle derivative with an *n*-butyloxy group, showed selective antiproliferation effects on oral cancer but not on normal oral cells. This investigation assessed for the first time the synergistic antiproliferation potential of cisplatin/SK2 in oral cancer cells. Cell viability assay at 24 h showed that a low dose of combined cisplatin/SK2 (10 μM/10 μg/mL) provided more antiproliferation than cisplatin or SK2 alone. Cisplatin/SK2 triggered also more apoptosis inductions in terms of subG1 accumulation, annexin V, pancaspase, and caspase 3/8/9 measurements. Moreover, cisplatin/SK2 provided more oxidative stress and DNA damage in oral cancer cells than independent treatments. Oxidative stress inhibitors rescued the cisplatin/SK2-induced antiproliferation and oxidative stress generation. Moreover, cisplatin/SK2 induced more antiproliferation, apoptosis, oxidative stress, and DNA damage in oral cancer cells than in normal oral cells (S-G). In conclusion, low-dose cisplatin/SK2 combined treatment promoted selective and synergistic antiproliferation in oral cancer cells depending on oxidative-stress-associated responses.

## 1. Introduction

Oral cancer is the top three cancer in Taiwan [1], and it also has a global impact [2]. It shows a low five-year survival rate when diagnosed late [3]. Typical oral cancer therapy includes surgery, chemotherapy, and radiation, which all have adverse side effects [4]. Moreover, chemotherapy and radiotherapy may provide resistance problems that limit the effectiveness of cancer therapy [5]. Recently, a low dose strategy was reported to overcome chemoresistance [6,7,8,9,10]. In an animal study, the low-dose anticancer drug doxorubicin overcomes the chemoresistance of patient-derived leukemia stem cells for prolonging survival [11]. Accordingly, low-dose drug treatments may overcome chemoresistance in cancer therapy.

In addition to dosage concerns [11], a combined treatment also provides an effective strategy to reduce resistance problems in cancer treatment [12,13,14,15,16,17,18]. For the complex causation and etiology of cancer, multiple therapies, including combinations of natural products, radiation, and anticancer agents, have been applied to oral cancer treatment [12,13]. Combination treatment may sensitize cancer cells to clinical drugs and reduce their potential adverse effects. For example, cisplatin is one of the effective anticancer drugs against oral, bladder, ovarian, leukemia, prostate, lung, and other cancers [19]. Cisplatin is commonly used for combined treatment applications with natural products [20], which may decrease the side effects of cisplatin. However, some studies for cisplatin combined treatments did not concern the responses of normal cells [21,22,23,24], and their potential side effects were not investigated. 

The dioxabicyclo [3.3.1]nonane core is a central and bioactive structure in many natural products. We previously reported a novel benzo-fused dioxabicyclo [3.3.1]nonane— namely, SK2, exhibiting selective antiproliferation effects against oral cancer cells with a mild adverse effect on normal cells [25]. SK2 also triggers oxidative stress, apoptosis, and DNA damage in oral cancer cells but these changes were not examined in normal oral cells [25]. An antioral cancer application of combined treatment with SK2 has not been reported thus far.

The aim of the present study is to investigate the synergistic effects of antiproliferation of a combined SK2/cisplatin treatment. This includes the pursuit of detailed mechanisms for possible synergistic antiproliferation.

## 2. Materials and Methods

### 2.1. SK2 Preparation and Inhibitors

SK2 (MW = 292.1059), IUPAC name: 6-*n*-butoxy-10-nitro-12,13-dioxa-11-azatricyclo [7.3.1.0^2,7^]trideca-2,4,6,10-tetraene, was prepared with >95% purity, as previously described [25], dissolved in DMSO (Sigma-Aldrich, St. Louis, MO, USA) for all experiments.

*N*-acetylcysteine (NAC) (Sigma-Aldrich, St. Louis, MO, USA) [26,27] and MitoTEMPO [28] (Cayman Chemical, Ann Arbor, MI, USA), the inhibitors of reactive oxygen species (ROS) and mitochondrial superoxide (MitoSOX), were dissolved in 1 x PBS and DMSO for stock preparation, respectively. Z-VAD-FMK (ZVAD) [29] (Selleckchem.com; Houston, TX, USA), a panapoptosis inhibitor, was dissolved in DMSO for stock preparation.

### 2.2. Cell Cultures and Cell Viability

ATCC (Manassas, VA, USA) oral cancer cell line (Ca9-22) and normal oral gingival epithelial Smulow–Glickman cell lines (S-G) [30,31,32] were used. Another oral cancer cell line (HSC-3) was derived from JCR Bank Cell lines (Ibaraki, Osaka, Japan). Standard medium mixed with 10% fetal bovine serum (FBS) and P/S antibiotics were cultured as instructed. Promega’s MTS kit (Madison, WI, USA) was chosen for determining cell viability [25].

### 2.3. Cell Cycle Assay

The cell cycle changes were analyzed by staining with 7-aminoactinomycin D (7AAD) (Biotium Inc., Hayward, CA, USA) [33]. In brief, 75% ethanol fixed cells were processed with PBS washing for 7AAD incubation (1 μg/mL, 30 min, 37 °C). Subsequently, cells were resuspended in PBS and studied with a flow cytometer (Accuri C6, BD Biosciences, Franklin Lakes, NJ, USA).

### 2.4. Annexin-V Apoptosis Assay

Annexin V is a common sensor for phosphatidylserine, which is detectable in the outer membrane of apoptotic cells. The apoptosis changes were detected by applying annexin V/7AAD kit (Strong Biotech Corp., Taipei, Taiwan) [25] as a user manual, i.e., annexin V-FITC (10 μg/mL) and 7AAD (1 μg/mL, 30 min, 37 °C). Subsequently, cells were mixed in PBS and studied with an Accuri C6 flow cytometer.

### 2.5. Caspase (Cas)-Apoptosis Assay

Cas signaling detection was firstly analyzed by a pancaspase kit (Abcam, Cambridge, UK), showing broad-spectrum responses to several caspases such as Cas-1 and 3 to 9 [25]. For specific intrinsic and extrinsic mediators and final executors of apoptotic Cas, the OncoImmunin kits (Gaithersburg, MD, USA) were chosen to detect Cas 3, Cas 8, and Cas 9 activities in a peptide-based reaction [25,34]. They were reacted with 10 μM specific peptides (PhiPhiLux-G1D2, CaspaLux8-L1D2, and CaspaLux9-M1D2) at 37 °C for 1 h. The activated Cas 3, Cas 8, and Cas 9 can digest these peptides to produce fluorescence for flow cytometry. Moreover, the Cas 3/7 activity was further validated by a luminescence-based Caspase-Glo^®^ 3/7 kit (Promega; Madison, WI, USA) [35].

### 2.6. ROS, MitoSOX, and Mitochondrial Membrane Potential (MMP) Assay

Cellular ROS can react with 2′,7′-dichlorodihydrofluorescein diacetate (H_2_DCF-DA) (Sigma-Aldrich; St. Louis, MO, USA) for flow cytometry [25], according to the user manual, i.e., 100 nM for 30 min. MitoSOX can react with MitoSOX™ Red (Molecular Probes, Invitrogen, Eugene, OR, USA) for flow cytometry [25], according to the user manual, i.e., 5 μM for 30 min. MMP can react with MitoProbe™ DiOC_2_ (3) (Invitrogen, San Diego, CA, USA) for flow cytometry [25], according to the user manual, i.e., 20 nM for 30 min.

### 2.7. γH2AX and 8-Hydroxy-2′-deoxyguanosine (8-OHdG) Assays

γH2AX antibody was applied at 1:50 (Santa Cruz Biotechnology, Santa Cruz, CA, USA). Then, it was coupled with Alexa Fluor 488-conjugated secondary antibody (Jackson Laboratory, Bar Harbor, ME, USA) for flow cytometry analysis. Similarly, an 8-OHdG-FITC antibody at 1:100 (Santa Cruz Biotechnology, Santa Cruz, CA, USA) was applied for flow cytometry analysis [25].

### 2.8. Statistics

In multi-comparison analysis, the Tukey post hoc test was performed by JMP 12 software (SAS Institute, Cary, NC, USA). The significance was indicated by not overlapping lower-cased letters. In contrast, data with overlapping lower-cased letters showed a nonsignificant difference. Triplicate experiments were performed and designated as means ± SDs.

## 3. Results

### 3.1. Cisplatin/SK2 (CDDP/SK2) Combined Treatment Causes Synergistic Antiproliferation in Oral Cancer Cells

**Figure 1:**
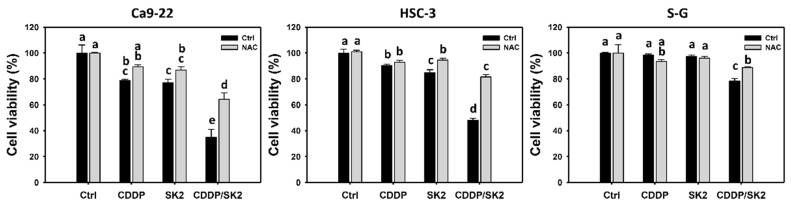
Cell viability for cisplatin (CDDP) and/or SK2. Following NAC preprocessing (10 mM for 1 h) or not, oral cancer (Ca9-22 and HSC-3) and normal oral (S-G) cells were processed with four treatments—control (0.1% DMSO), CDDP (10 μM), SK2 (10 μg/mL), and CDDP/SK2 (10 μM/10 μg/mL)—for 24 h and assessed by an MTS assay. For multi-comparisons, data without overlapping letters differ significantly (*p* < 0.05). The highest value is marked with a, and others are shown in descending order. The data are shown as means ± SD (*n* = 3). In the example of Ca9-22 cells, the cell viabilities between the control (black) and NAC (gray) for CDDP/SK2 (e vs. d), showing nonoverlapping characters, differed significantly. Similarly, the control (black) and CDDP/SK2 (black) (a vs. e) were significantly different. In contrast, the controls (black) for CDDP and SK2 (bc vs. c) did not differ significantly.

A formazan-based MTS kit is commonly used to detect cell proliferation. Based on a 24 h MTS assay, a combined cisplatin/SK2 treatment decreased cell viability for 34.91% and 48% of oral cancer cells (Ca9-22 and HSC-3, respectively) than an independent, separate treatment (cisplatin (78.74% and 90.37%) or SK2 (76.96% and 84.84%) alone) (Figure 1). Moreover, cisplatin/SK2 combined treatment decreased cell viability to a greater extent in oral cancer cells (Ca9-22 and HSC-3) (34.91% and 48%) than in normal cells (S-G) (78.43%). 

To assess the effects of oxidative stress, the ROS inhibitor NAC was applied. The antiproliferation of cisplatin/SK2 single and combined treatments was suppressed by NAC, suggesting that synergistic antiproliferation of cisplatin/SK2 was mediated by oxidative stress.

### 3.2. Cisplatin/SK2 Causes Synergistic subG1 Accumulation in Oral Cancer Cells

**Figure 2:**
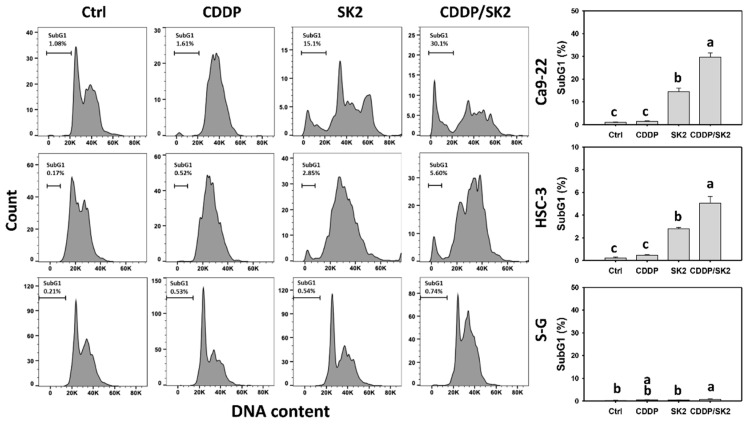
Cell cycle assessment for cisplatin (CDDP) and/or SK2. Oral cancer (Ca9-22 and HSC-3) and normal (S-G) cells were processed with four treatments—control (0.1% DMSO), CDDP (10 μM), SK2 (10 μg/mL), and CDDP/SK2 (10 μM/10 μg/mL)—for 24 h and were assessed by a cell cycle assay. For multi-comparisons, data without overlapping characters differ significantly (*p* < 0.05). The highest value is marked with a, and others are shown in descending order. The data are provided as means ± SD (*n* = 3). In the example of Ca9-22 and HSC-3 cells, the subG1 (%) for control, SK2, and CDDP/SK2 (c, b, a) differed significantly. In contrast, the controls for CDDP showing the overlapping character “c” did not differ significantly.

To assess the involvement of apoptosis in the antiproliferation of SK2, the subG1 status in cell cycle analysis was observed. The oral cell cycle patterns of four treatments—the control, cisplatin, SK2, and cisplatin/SK2—were demonstrated for oral cancer cells (Ca9-22 and HSC-3 cells) and normal cells (S-G) (Figure 2). A combined cisplatin/SK2 showed higher sub-G1 (%) than independent treatments (cisplatin or SK2) and control. Moreover, a combined cisplatin/SK2 increased more subG1 (%) in oral cancer cells than in normal cells (S-G), indicating that cisplatin/SK2 induces selectively subG1 accumulation in oral cancer cells indicating apoptosis.

### 3.3. Cisplatin/SK2 Causes Synergistic Annexin-V-Detected Apoptosis in Oral Cancer Cells

**Figure 3:**
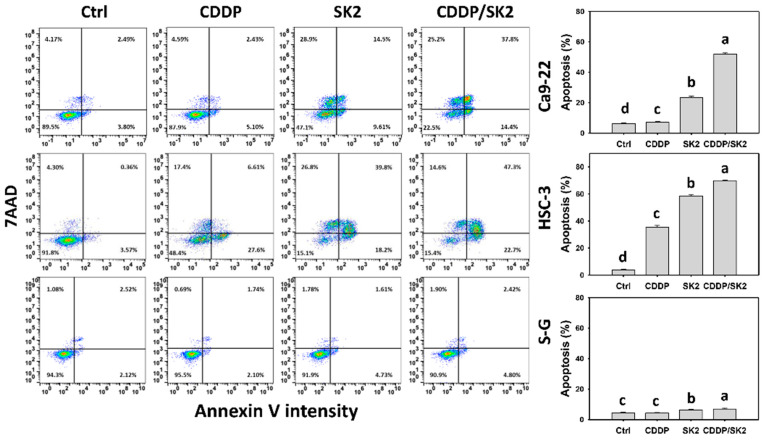
Annexin-V assessment for cisplatin (CDDP) and/or SK2. Oral cancer (Ca9-22 and HSC-3) and normal (S-G) cells were processed with four treatments—control (0.1% DMSO), CDDP (10 μM), SK2 (10 μg/mL), and CDDP/SK2 (10 μM/10 μg/mL)—for 24 h and assessed by an annexin-V assay. The annexin-V-positive (%) is defined as apoptosis (%). For multi-comparisons, data without overlapping characters differ significantly (*p* < 0.05). The highest value is marked with a, and others are shown in descending order. Data are shown as means ± SD (*n* = 3). In the example of Ca9-22 cells, the apoptosis (%) for control, CDDP, SK2, and CDDP/SK2 (d, c, b, a), showing nonoverlapping characters, differed significantly. For S-G cells, the controls and CDDP showing overlapping character “c” did not differ significantly.

The apoptosis potential of subG1 accumulation in Figure 2 was further examined by an annexin-V assay, which detected the phosphatidylserine in the outer plasma membrane of apoptotic cells. Cisplatin/SK2 combined treatment displayed higher apoptosis (%) in oral cancer cells (Ca9-22 and HSC-3) than the independent treatments (cisplatin or SK2) and control (Figure 3). Moreover, a combined cisplatin/SK2 decreased apoptosis (annexin V) in oral cancer cells more than in normal cells (S-G), indicating that cisplatin/SK2 induces selective apoptosis in oral cancer cells.

### 3.4. Cisplatin/SK2 Shows Synergistic Apoptosis (Caspase Activation) in Oral Cancer Cells

**Figure 4:**
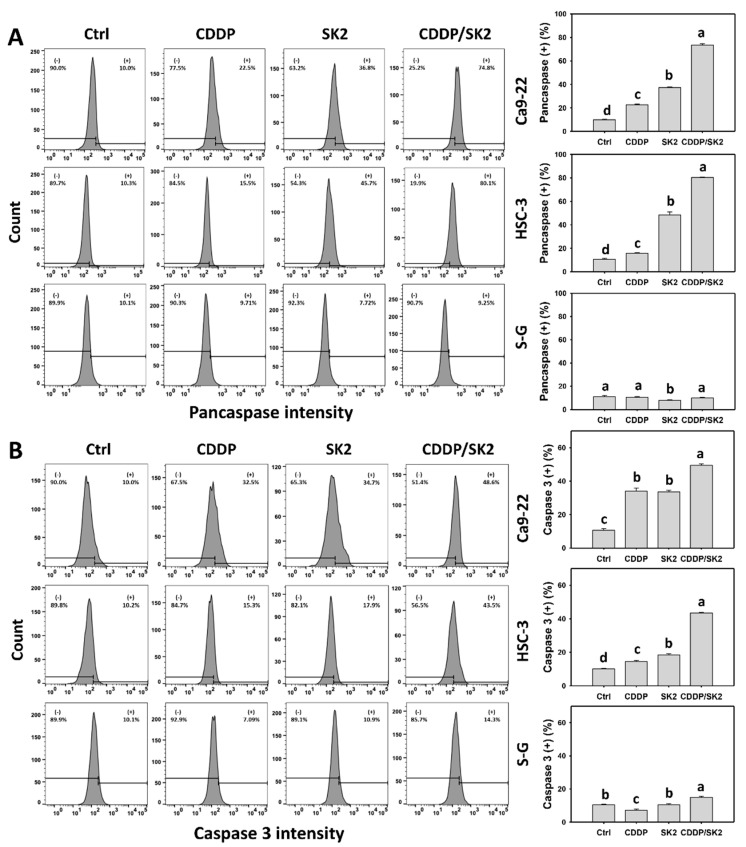

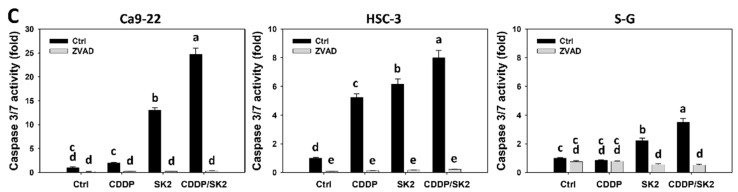
Flow cytometry (pancaspase and Cas 3) and luminescence (Cas 3/7) assays for cisplatin (CDDP) and/or SK2: (**A**,**B**) pancaspase and Cas 3 flow cytometry: (+) indicates pancaspase or Cas 3-positive (%); (**C**) Cas 3/7 luminescent assay. Following the ZVAD preprocessing (2 μM for 2 h) or not, oral cancer (Ca9-22 and HSC-3) and normal (S-G) cells were processed with four treatments—control (0.1% DMSO), CDDP (10 μM), SK2 (10 μg/mL), and CDDP/SK2 (10 μM/10 μg/mL)—for 24 h and assessed by flow cytometry and luminescence assays. (+) is defined as high intensity of Cas 3. For multi-comparisons, data without overlapping characters differ significantly (*p* < 0.05). The highest value is marked with a, and others are shown in descending order. The data are shown as means ± SD (*n* = 3). In the example of Ca9-22 cells (Figure 4C), the Cas 3/7 activity between the control (black) and ZVAD (gray) for CDDP (c vs. d), SK2 (b vs. d), CDDP/SK2 (a vs. d), showing nonoverlapping characters, differed significantly. Similarly, the control, SK2, and CDDP/SK2 (cd, b, a) varied significantly. In contrast, the control and CDDP (cd vs. c) showing the overlapping character “c” did not differ significantly.

Apoptosis is triggered by the signaling of several caspases. The caspase assay was further applied to examine the apoptosis potential of subG1 accumulation in Figure 2 in terms of flow cytometry (pancaspase and Cas 3) and luminescence detection. A cisplatin/SK2 combined treatment displayed higher pancaspase (+) (%) in oral cancer cells (Ca9-22 and HSC-3) than the independent treatments (cisplatin or SK2) and control (Figure 4A). Moreover, a combined cisplatin/SK2 increased more pancaspase (+) (%) in oral cancer cells than in normal cells (S-G), indicating that cisplatin/SK2 induces selective pancaspase activation in oral cancer cells.

Since the pancaspase assay provides a broad-spectrum detection for several caspases (Cas 1 and Cas 3 to 9) [25], a specific Cas 3 assay was performed. In terms of flow cytometry, a combined cisplatin/SK2 treatment displayed higher Cas 3 (+) in oral cancer cells than in independent treatments (Figure 4B). Moreover, a combined cisplatin/SK2 increased more Cas 3 (+) (%) in oral cancer cells than in normal cells (S-G). 

Similarly, luminescence detection of Cas 3/7 (Figure 4C) showed the same tendency as flow cytometry (Figure 4B). The Cas 3 induction of cisplatin/SK2 combined treatment was suppressed by ZVAD, suggesting that caspase signaling was triggered by apoptosis. Moreover, cisplatin/SK2 combined treatment induced more Cas 3/7 activity in oral cancer cells than in normal cells (S-G), suggesting that cisplatin/SK2 induces selective caspase activation in oral cancer cells (Figure 4C).

### 3.5. Cisplatin/SK2 Shows Synergistic Induction of Extrinsic and Intrinsic Apoptosis in Oral Cancer Cells

**Figure 5:**
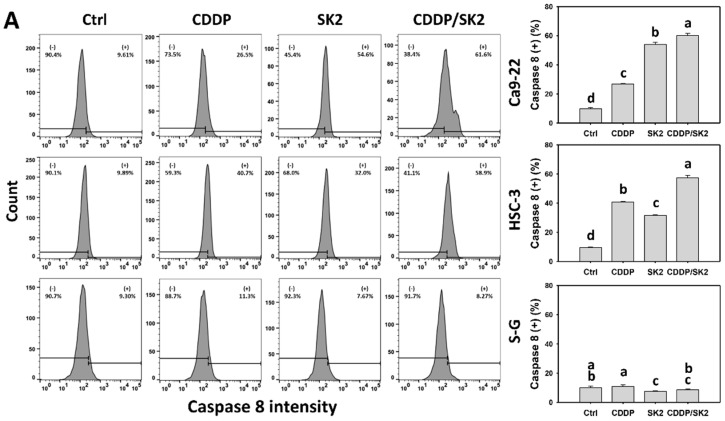

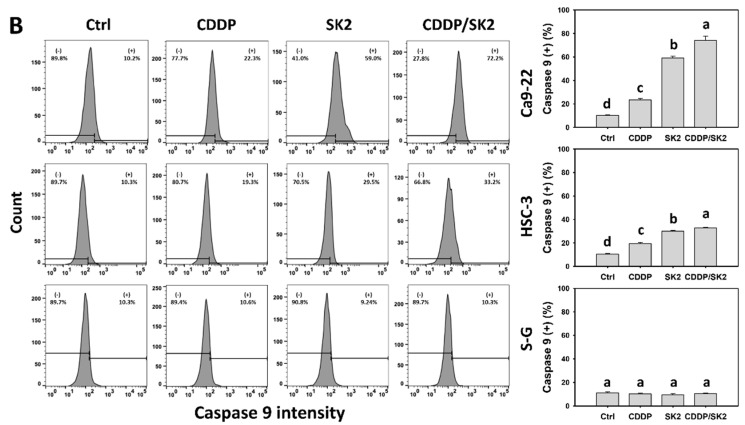
Cas 8 and Cas 9 assays for cisplatin (CDDP) and/or SK2: (**A**) Cas 9 flow cytometry; (**B**) Cas 9 flow cytometry. Oral cancer (Ca9-22 and HSC-3) and normal (S-G) cells were processed with four treatments—control (0.1% DMSO), CDDP (10 μM), SK2 (10 μg/mL), and CDDP/SK2 (10 μM/10 μg/mL)—for 24 h and assessed by Cas 8 and Cas 9 assays. (+) is defined as high intensity of Cas 8 and Cas 9. For multi-comparisons, data without overlapping characters differ significantly (*p* < 0.05). The highest value is marked with a, and others are provided in descending order. The data are shown as mean ± SD (*n* = 3). In the example of Ca9-22 cells (Figure 5B), Cas 9 (+) (%) for control, CDDP, SK2, and CDDP/SK2 (d, c, b, a), showing nonoverlapping characters, differed significantly.

The extrinsic and intrinsic apoptosis signaling was mainly triggered by Cas 8 and Cas 9, and they were further examined by Cas 8 and Cas 9 flow cytometry, respectively. A combined cisplatin/SK2 showed higher Cas 8 and Cas 9 (+) (%) in oral cancer cells (Ca9-22 and HSC-3) than the independent treatments (cisplatin or SK2) and control (Figure 5A and 5B). Moreover, a combined cisplatin/SK2 increased more Cas 8 and Cas 9 (+) (%) in oral cancer cells than in normal cells (S-G), indicating cisplatin/SK2 induces selective extrinsic and intrinsic caspase activations in oral cancer cells.

### 3.6. Cisplatin/SK2 Combined Treatment Shows Synergistic Induction of ROS in Oral Cancer Cells

**Figure 6:**
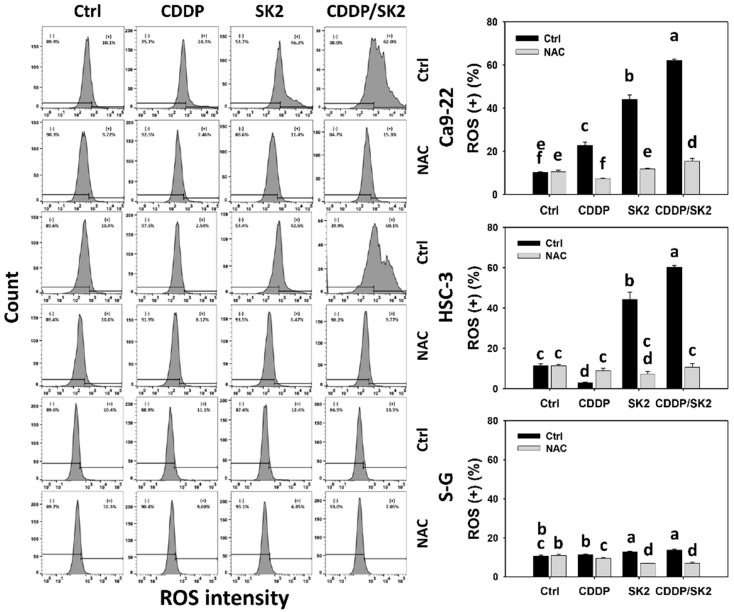
ROS assay for cisplatin (CDDP) and/or SK2. Following the NAC preprocessing (10 mM for 1 h) or not, oral cancer (Ca9-22 and HSC-3) and normal (S-G) cells were processed with four treatments—control (0.1% DMSO), CDDP (10 μM), SK2 (10 μg/mL), and CDDP/SK2 (10 μM/10 μg/mL)—for 24 h and assessed by ROS assays. (+) is defined as high intensity of ROS. For multi-comparisons, data without overlapped characters differ significantly (*p* < 0.05). The highest value is marked with a, and others are shown in descending order. The data are shown in the means ± SD (*n* = 3). In the example of Ca9-22 cells, the ROS (+) (%) between the control (black) and NAC (gray) for CDDP (c vs. f), SK2 (b vs. e), CDDP/SK2 (a vs. d) differed significantly. Similarly, the control, CDDP, SK2, and CDDP/SK2 (ef, c, b, a) were significantly different. In contrast, the control and NAC (ef vs. e) showing overlapping character “e” did not differ significantly.

Since NAC rescued the synergistic antiproliferation of cisplatin/SK2 in oral cancer cells (Figure 1), oxidative stress was further examined by ROS flow cytometry. Cisplatin/SK2 combined treatment displayed higher ROS (+) (%) in oral cancer cells (Ca9-22 and HSC-3) than the independent treatments (cisplatin or SK2) and control (Figure 6). Moreover, cisplatin/SK2 combined treatment induced more ROS (+) (%) in oral cancer cells than in normal cells (S-G), suggesting that cisplatin/SK2 induces selective ROS generation in oral cancer cells. Moreover, a combined cisplatin/SK2 treatment increased more ROS (+) (%) in oral cancer cells than in normal cells (S-G), indicating that cisplatin/SK2 induced the generation of more ROS in oral cancer cells. 

To assess the involvement of oxidative stress, the ROS inhibitor NAC was applied. The ROS (+) (%) of cisplatin/SK2 separate and combined treatments was suppressed by NAC, suggesting that synergistic ROS generation of cisplatin/SK2 is mediated by oxidative stress.

### 3.7. Cisplatin/SK2 Shows Synergistic Induction of MitoSOX in Oral Cancer Cells

**Figure 7:**
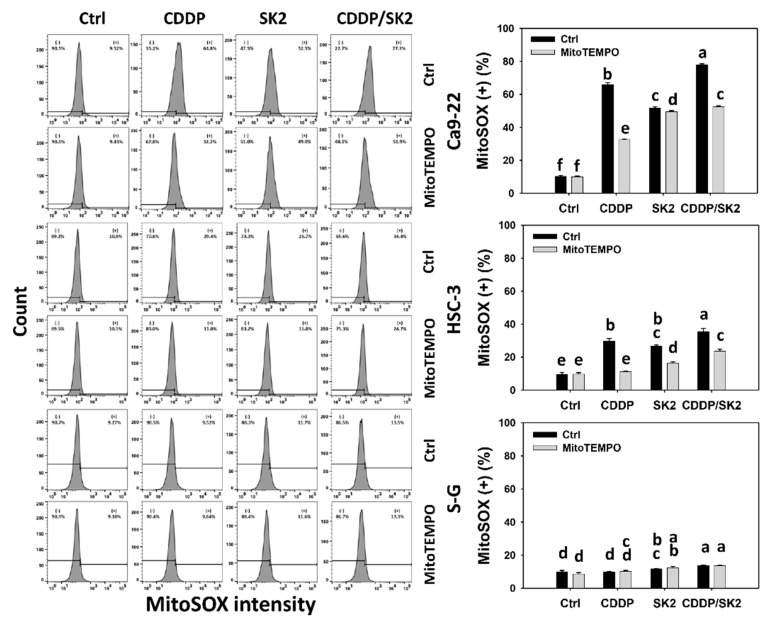
MitoSOX assay for cisplatin (CDDP) and/or SK2. Following the MitoTEMPO preprocessing (50 μM for 1 h) or not, oral cancer (Ca9-22 and HSC-3) and normal (S-G) cells were processed with four treatments—control (0.1% DMSO), CDDP (10 μM), SK2 (10 μg/mL), and CDDP/SK2 (10 μM/10 μg/mL)—for 24 h and assessed by MitoSOX assays. (+) is defined as high intensity of MitoSOX. For multi-comparisons, data without overlapping characters differ significantly (*p* < 0.05). The highest value is marked by a, and others are shown in descending order. The data are shown as means ± SD (*n* = 3). For the example of Ca9-22 cells, the MitoSOX (+) (%) between the control (black) and MitoTEMPO (gray) for CDDP (b vs. e), SK2 (c vs. d), CDDP/SK2 (a vs. c), showing nonoverlapping characters, differed significantly. Similarly, the control, CDDP, SK2, and CDDP/SK2 (f, b, c, a) differed significantly. In contrast, the control and MitoTEMPO showing an overlapping character “f” did not differ significantly.

As described (Figure 1), NAC rescued the synergistic antiproliferation of cisplatin/SK2 in oral cancer cells. In addition to cellular oxidative stress, NAC can also suppress the generation of mitochondrial reactive species such as mitochondrial superoxide (MitoSOX) [36]. Accordingly, the oxidative stress of mitochondria was further examined by MitoSOX flow cytometry. Cisplatin/SK2 combined treatment displayed a higher MitoSOX (+) (%) in oral cancer cells (Ca9-22 and HSC-3) than independent treatments (cisplatin or SK2) and control (Figure 7). Moreover, a combined cisplatin/SK2 increased more MitoSOX (+) (%) in oral cancer cells than in normal cells (S-G), indicating cisplatin/SK2 induces selective MitoSOX generation in oral cancer cells.

To assess the involvement of oxidative stress of mitochondria, the MitoSOX inhibitor MitoTEMPO was applied. The MitoSOX (+) (%) of cisplatin/SK2 single and combined treatments was suppressed by MitoTEMPO, suggesting that synergistic MitoSOX generation of cisplatin/SK2 is mediated by oxidative stress.

### 3.8. Cisplatin/SK2 Shows Synergistic MMP Destruction of Oral Cancer Cells

**Figure 8:**
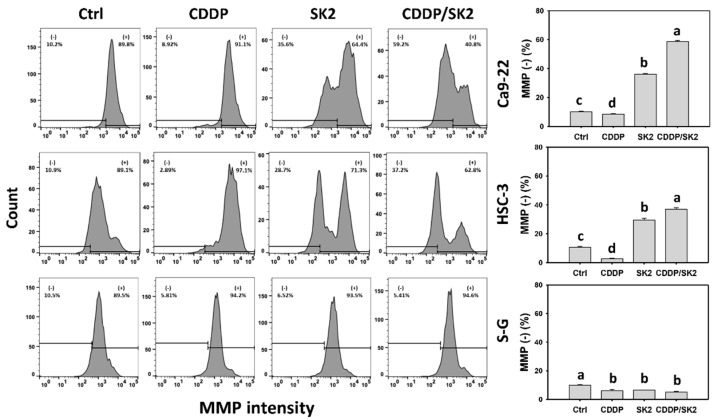
MMP assays for cisplatin (CDDP) and/or SK2. Oral cancer (Ca9-22 and HSC-3) and normal (S-G) cells were treated in four ways—control (0.1% DMSO), CDDP (10 μM), SK2 (10 μg/mL), and CDDP/SK2 (10 μM/10 μg/mL)—for 24 h and assessed by an MMP assay; (−) is defined as low level of MMP. For multi-comparisons, data without overlapping characters differ significantly (*p* < 0.05). The highest value is marked with a, and others are shown in descending order. The data are shown as means ± SD (*n* = 3). In the example of Ca9-22 cells, the MMP (−) (%) for control, CDDP, SK2, and CDDP/SK2 (c, d, b, a), showing nonoverlapping characters, differed significantly.

MMP destruction is associated with oxidative stress [37]. Since NAC rescued the synergistic antiproliferation of cisplatin/SK2 in oral cancer cells (Figure 1), oxidative stress was further examined by MMP flow cytometry. Cisplatin/SK2 combined treatment displayed higher MMP (−) (%) in oral cancer cells (Ca9-22 and HSC-3) than in independent treatments (cisplatin or SK2) and control (Figure 8). Moreover, a combined cisplatin/SK2 increased more MMP (−) (%) in oral cancer cells than in normal cells (S-G), indicating that cisplatin/SK2 treatment induces selective MMP destruction in oral cancer cells.

### 3.9. Cisplatin/SK2 Shows Synergistic Induction of γH2AX and 8-OHdG in Oral Cancer Cells

**Figure 9:**
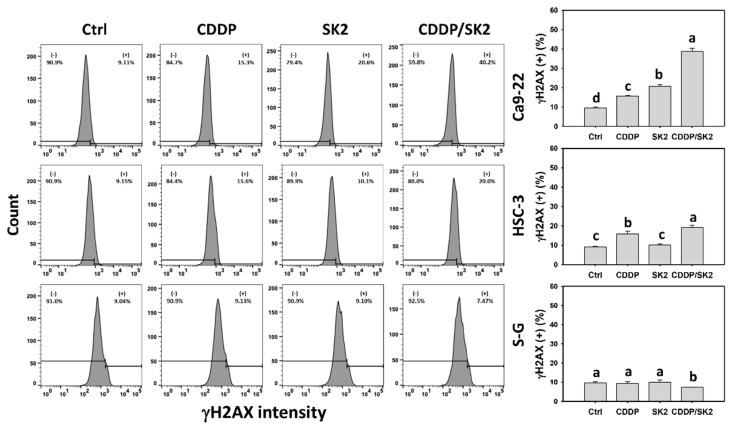
γH2AX assays for cisplatin (CDDP) and/or SK2. Oral cancer (Ca9-22 and HSC-3) and normal (S-G) cells were treated in four different ways—control (0.1% DMSO), CDDP (10 μM), SK2 (10 μg/mL), and CDDP/SK2 (10 μM/10 μg/mL)—for 24 h and assessed by γH2AX assays. (+) is defined as high intensity of MMP. For multi-comparisons, data without overlapping characters differ significantly (*p* < 0.05). The highest value is marked with a, and others are shown in descending order. The data are presented as means ± SD (*n* = 3). In the example of Ca9-22 cells, the γH2AX (+) (%) for control, CDDP, SK2, and CDDP/SK2 (d, c, b, a), showing nonoverlapping characters, differed significantly.

**Figure 10:**
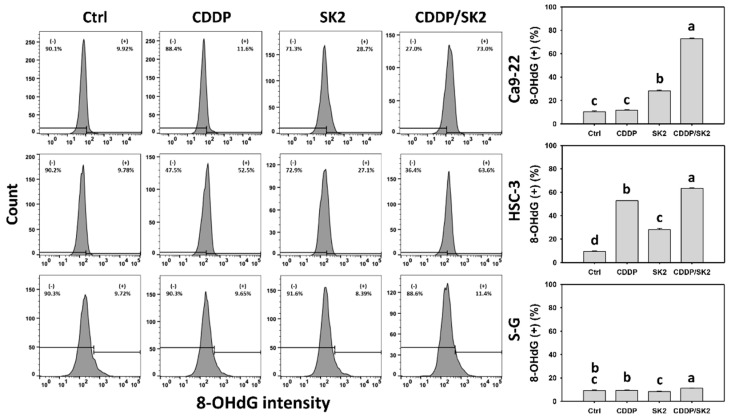
The 8-OHdG assays for cisplatin (CDDP) and/or SK2. Oral cancer (Ca9-22 and HSC-3) and normal (S-G) cells were treated in four ways—control (0.1% DMSO), CDDP (10 μM), SK2 (10 μg/mL), and CDDP/SK2 (10 μM/10 μg/mL)—for 24 h and assessed by 8-OHdG assay; (+) is defined as high intensity of MMP. For multi-comparisons, data without overlapping characters differ significantly (*p* < 0.05). The highest value is marked with a, and others are shown in descending order. The data are shown as means ± SD (*n* = 3). In the example of Ca9-22 cells, 8-OHdG (+) (%) for control, SK2, and CDDP/SK2 (c, b, a), showing nonoverlapping characters, differed significantly. In contrast, the control and CDDP (c) showing overlapping characters did not differ significantly.

The DNA damage effects of cisplatin and/or SK2-induced oxidative stress were examined. Cisplatin/SK2 combined treatment displayed greater DNA damages such as γH2AX and 8-OHdG (+) (%) in oral cancer cells (Ca9-22 and HSC-3) than in independent treatments (cisplatin or SK2) and control (Figure 9 and Figure 10).

## 4. Discussion

The present study evaluated the modulating proliferation ability of SK2–cisplatin combined treatment in oral cancer cells. The drug cytotoxicity of this combined treatment was examined in normal oral cells. In addition to cell viability, the responses of oxidative stress, apoptosis, and DNA damage were investigated and compared between oral cancer and normal oral cells following SK2 and/or cisplatin treatments.

Natural products and synthetic chemicals can sensitize oral cancer cells for chemotherapy and radiotherapy [35,38,39]. For example, the combined treatment of ethyl acetate extract of *Nepenthes ventricosa* x *maxima* (EANV)/cisplatin synergistically inhibited the proliferation of oral cancer cells [38]. Sulfonyl chromen-4-ones/UVC [35] and sulfonyl chromen-4-ones/X-ray [40] can synergistically inhibit proliferation against oral cancer cells. A combined treatment of cordycepin/cisplatin synergistically enhanced apoptosis in oral cancer OC3 cells [39]. Combined treatments may, therefore, effectively improve oral cancer therapies.

A dioxabicyclo [3.3.1]nonane core commonly appears in several natural products, such as in the marine algal toxins azaspiracids [41], epicoconigrone A [42], and epicoccolide A [43]. However, the modulating antiproliferation ability of dioxabicyclo [3.3.1]nonane derivatives has rarely been investigated. Our previous study first developed a novel chemical with a dioxabicyclo [3.3.1]nonane core (SK2) and showed the antiproliferation effect on oral cancer cells [25]. Nevertheless, SK2-induced oxidative stress, DNA damage, and apoptosis have not been assessed in normal oral cells thus far. Moreover, to date, the combined treatment for most chemicals with a dioxabicyclo [3.3.1]nonane core has rarely been reported.

Several selective killing agents have been reported, which show higher antiproliferation ability in cancer cells than in normal cells [44,45,46,47]. Combining the selective killing agents with clinical drugs may potentially avoid the side effects of anticancer drugs. For example, cisplatin and FK228 (romidepsin, a histone deacetylase inhibitor) [48] show the selective killing of breast cancer cells with low cytotoxicity to normal breast cells and cause synergistic antiproliferation in breast cancer cells [49]. Accordingly, these combined treatments may exhibit synergistic and selective antiproliferation against cancer cells. SK2 is also a selective killing agent [25], inhibiting the proliferation of oral cancer cells more pronouncedly than those of normal cells. As expected, the present study first demonstrated that a structure with dioxabicyclo [3.3.1]nonane core such as SK2 could sensitize the cisplatin response to oral cancer cells (Figure 1). It warrants a detailed assessment of the synergistic antiproliferation for combined treatment of SK2 and other clinical drugs such as curcumin, doxorubicin, and 5-fluorouracil in the future.

Several studies have reported synergistic enhancement of oxidative stress in combined treatments could increase antiproliferation. For example, cisplatin and FK228 combined treatment cooperatively induces ROS levels and causes synergistic antiproliferation in breast cancer cells [49]. Salinomycin is a monocarboxylic polyether ionophore that shows synergistic ROS induction combined with resveratrol, causing synergistic antiproliferation in breast cancer cells [50]. A combined treatment of dihydroartemisinin and cisplatin provides a synergistic effect of ROS generation and antiproliferation in pancreatic cancer cells [17]. Additionally, sulfonyl chromen-4-ones/X-ray induces higher ROS generation and antiproliferation in oral cancer cells [40].

SK2 contains an N-O bond and NO_2_ group, showing free radical potential [51,52]. This radical character also demonstrates oxidative stress in oral cancer cells [25], such as the generation of ROS and MitoSOX and MMP destruction. However, the role of oxidative stress for SK2 was not examined [25]. In contrast, in the present study, all changes in oxidative stress, DNA damage, and apoptosis of SK2 were examined in normal oral cells in detail. The combined treatment of SK2/cisplatin showed synergistic ROS and MitoSOX generation in oral cancer cells but not in normal oral cells (Figure 6 and Figure 7). NAC pretreatment suppresses synergistic antiproliferation (Figure 1) and the ROS induction (Figure 6) of SK2/cisplatin in oral cancer cells. Similarly, MitoTEMPO pretreatment suppresses synergistic MitoSOX induction (Figure 7). These results confirm the role of oxidative stress in synergistic antiproliferation and oxidative stress of SK2/cisplatin. Therefore, synergistic oxidative stress contributes to synergistic antiproliferation in SK2/cisplatin combined treatment of oral cancer cells. 

Moreover, several anticancer agents generate oxidative stress, improving apoptosis [53,54,55] and DNA damage [27,54]. Except for synergistic oxidative stress, the combined treatments show synergistic apoptosis and DNA damage, such as cisplatin/FK228 in breast cancer [49], salinomycin/resveratrol in breast cancer [50], dihydroartemisinin/cisplatin in pancreatic cancer [17], and sulfonyl chromen-4-ones/X-ray in oral cancer cells. Similarly, SK2/cisplatin combined treatment show synergistic intrinsic and extrinsic apoptosis (Figure 5) and DNA damage (γH2AX and 8-OHdG) (Figure 9 and Figure 10) in oral cancer cells but not in normal oral cells. Additionally, a combined SK2/cisplatin treatment shows synergistic inductions of annexin V, pancaspase, Cas 3/8/9, and Cas 3/7 (Figure 3 and Figure 4) in oral cancer cells but not in normal oral cells. Therefore, it is likely oxidative stress that contributes to synergistic apoptosis and DNA damage in SK2/cisplatin combined treatment of oral cancer cells.

## 5. Conclusions

Our previous research showed that the nitrated [6,6,6]tricycle-derived compound (SK2) exhibited a selective antiproliferation ability in oral cancer cells [25]. However, its potential combined treatment with clinical drugs was not reported. Taking cisplatin as an example, the combined treatment of SK2 was examined in the present study. Combined treatment with cisplatin/SK2 shows synergistic and selective antiproliferation in oral cancer cells but not in normal oral cells. Moreover, cisplatin/SK2 shows a synergistic induction of oxidative stress, caspase-dependent apoptosis, and DNA damage in oral cancer cells at low concentrations of cisplatin and SK2. This finding suggests that SK2 has the potential to be applied in combined treatment with clinical drugs.

## Data Availability

All data are made available in this article.
